# Malnutrition in Early Life, Socio-demographic and Self-reported Appetite in Adulthood in Chongqing, China

**Published:** 2017-03

**Authors:** Xianglong XU, Lingli LIU, Yunshuang RAO, Lu GUO, Yong ZHAO

**Affiliations:** 1. School of Public Health and Management, Chongqing Medical University, Chongqing, China; 2. Research Center for Medicine and Social Development, Chongqing Medical University, Chongqing, China; 3. The Innovation Center for Social Risk Governance in Health, Chongqing Medical University, Chongqing, China; 4. School of Nursing, Chongqing Medical University, Chongqing, China; 5. Children’s Hospital of Chongqing Medical University, Chongqing Medical University, Chongqing, China

## Dear Editor-in-Chief

The Chinese famine between 1959 and 1961 is the largest food crisis in human history resulted in approximately 30 million fatalities (1). Before the 1959–1961 famine, China’s grain output experienced steady growth and reached a peak of 200 million tons in 1958 (2). The estimated daily per capita availability of food energy during 1959–61 decreased considerably, falling well below average food energy requirements (about 2100 calories). The worst case occurred in 1960, with only 1500 calories (3). National death rates were 14, 25, and 14 per thousand for the years of 1959, 1960, and 1961, respectively, while the average death rate for 1956–1958 was only about 11 per thousand. Fertility rates also dropped sharply in the years 1959–1961 (4). Food availability was much worse in rural areas than urban areas (5). Exposure to malnutrition or other adverse environments in the fetal period and early childhood exerts significant lasting effects on diabetes (6), metabolic syndrome (1) in adulthood.

Appetite plays an important role in every aspect of life. Appetite is an instinctive physical desire, especially one for food or drink. Appetite is regulated by a close interplay between the digestive tract, adipose tissue, and the brain, has a relationship with emotion, psychological factors and every individual’s behaviors (7). The developmental origins hypothesis proposes that under nutrition in early life is associated with an increased risk of disease in adulthood (8). This study aimed to explore malnutrition in early fife, socio-demographic and self-reported appetite in adulthood.

This cross-sectional study adopted multi-stage stratified random sampling was employed in selecting the participants of this study. The effects of famine were much more devastating in rural areas than in urban areas (4). Overall, 1250 participants in Chongqing, China were included in the survey in July 2009. Among them, the response rate was 1230 (98.4%); six responses were disregarded because of missing data, resulting in a final sample of 1224 in the analysis.

This study was approved by the Ethics Committee of the Chongqing Medical University. All participants submitted written informed consent.

The participants were categorized into three groups based on birth date: 1) childhood exposure: 470 (1–3 yr before the famine (1956–1958)); 2) fetal exposure: 299 (three years during the famine (1959–1961)); 3) non-exposure: 455 (1–3 yr after the famine (1962–1964)). Appetite was self-reported measured. Appetite in this study focuses on “desire to eat”.

Logistic regression model to predict factors affect poor appetite in adulthood (Table 1).

**Table 1:** Factors associated with poor appetite

**Predictors**	**Model1**	**Model2**	**Model3**
	OR, 95% CI	OR, 95% CI	OR, 95% CI
**Educational level**			
Secondary Education vs. Basic Education		0.596(0.398,0.894)^*^	
Higher Education vs. Basic Education		0.693(0.450,1.065)	
**Observance of life routine**			
Sometimes vs. Seldom		1.447(1.086,1.926)*	1.501(1.126,2.001)*
Usually vs. Seldom		0.828(0.470,1.457)	0.906(0.515,1.593)
**Sleep status**			
Average vs. Poor		0.834(0.507,1.372)	0.844(0.512,1.390)
Good vs. Poor		0.102(0.061,0.171)	0.102(0.061,0.172)*
**Family relationships**			
Average vs. Poor		0.605(0.175,2.094)	0.583(0.163,2.089)
Harmonious vs. Poor		0.411(0.121,1.401)	0.379(0.107,1.334)
**Current living conditions**			
Average vs. Unsatisfactory		0.709(0.355,1.418)	0.697(0.348,1.396)
Satisfactory vs. Unsatisfactory		0.390(0.194,0.784)*	0.376(0.187,0.757)*
**Timing of exposure to famine**			
Childhood exposure vs. Non-exposure	1.517(1.133,2.031)^*^		1.775(1.251,2.518)*
Fetal exposure vs. Non-exposure	1.496(1.155,1.939)^*^		1.574(1.156,2.144)*

Note: 1) Abbreviation: CI confidence intervals, OR odds ratio.

2) ^*^statistically significant (*P*<0.05).

Fourteen factors were considered in the modeling of to predict factors affect poor appetite in adulthood: timing of exposure to famine, gender, educational level, marital status, job conditions, average monthly income, smoking, alcohol drinking, sports frequency, observance of life routine, sleep status, relationships with family, current living conditions, feeding procedure). Moreover, we established three model to analysis the factors associated with poor appetite, respectively model 1: adjusted for Timing of exposure to famine; model 2: adjusted for all fourteen factors, excepted Timing of exposure to famine; model 3: adjusted for all fourteen factors. In model 3, people sometimes have observance of life routine are more likely to have poor appetite than people of seldom have observance of life routine (OR = 1.501, 95% CI (1.126–2.001). Persons with good sleep are less likely to have poor appetite (OR=0.102, 95% CI (0.061–0.172). Participants satisfied with current living conditions were less likely to have poor appetite (OR=0.376, 95% CI (0.187–0.757). People exposed to famine when childhood (OR=1.775, 95% CI [1.251–2.518]) and people exposed to famine when fetal (OR=1.574, 95% CI [1.156–2.144]) was more likely to have poor appetite than non-exposed to famine,

Childhood and fetal exposure to famine were more likely to have poor self-reported appetite in adulthood. Early life environment may affect appetite in adult life. Regular daily life, sleep, and current living conditions affect appetite in adulthood. This research provides guidance for building prevention on appetite to adults in China as well as a contribution to enhancing understanding the pathogenesis of poor appetite.
